# Hospitals and the Spirit Tax

**Published:** 1915-07-31

**Authors:** 


### HOSPITALS AND THE SPIRIT TAX.

### Where the British Medical Association's Case Fails.

In the official organ of the British Medical Association of July 24 is published what is termed "a brief statement of the facts" relative to the Association's attitude towards the proposal of the Government to relieve hospitals of the heavy burden imposed on them by the spirit duties. This statement, which might be better described as an apology, is obviously directed to the criticisms of the Association's policy which have recently appeared in The Hospital; it does not differ in any material respect from the letter from the chairman of the Parliamentary Sub-Section of the Association which it was our pleasure to publish last week, and therefore a reply to the explanation of the *British Medical Journal* will also serve as an answer to the letter. There is, however, one point on which it is desirable to reassure Mr. Harman; that is, that our leading article of June 17 was written with a full knowledge of all the essential features of the negotiations which had taken place up to the close of the Report stage of the Finance Bill. The Government clause had been dropped and a provisional promise had been made that a clause embodying a similar principle would be introduced on a subsequent occasion; but, as we showed last week, the Chancellor of the Exchequer may be prevented from carrying out his good intentions either from inability to find the necessary time or because of the refusal of the temperance party to accept the principle. It would, by the way, be interesting to know to what extent the misconceptions of the temperance party with respect to the proposal are due to the representations made in the House of Commons on behalf of the Association. It has been shown by the pharmaceutist to the London Hospital, in a letter to this journal, that those representations were based upon a very inadequate knowledge of the conditions governing the manufacture and dispensing of medicines in voluntary institutions, and as it will be essential to secure the support of the temperance party before Mr. McKenna's provisional promise can be carried out, it is absolutely necessary that their misconceptions should be removed; and we suggest that the proper authority to remove them is the British Medical Association.

Examining the "brief statement of facts" published by our contemporary, we find the observation that no "warning" had been given that such a clause would appear in the Bill. Now on April 21 Mr. Lloyd George stated that he was considering the suggestion of giving an exemption from spirit duty to hospitals, and the clause which presumably reflected the mental efforts he had exerted in this connection did not appear in the Parliamentary papers until the third week in June; surely an Association which places such implicit confidence in the promises of Ministers ought to regard an intimation given seven clear weeks before the event as a sufficient warning that something might be done. Possibly the reply will be that the Chancellor's statement was too indefinite; we would therefore draw the attention of the Association to the official report of the House of Commons debates on May 19, where it will be found that on that date Mr. Bridgeman asked the Chancellor of the Exchequer "how he proposes to carry out the promise made to the hon. member for Oswestry that he would exonerate from duty spirit used by hospitals for tinctures or other hospital purposes," and Mr. Lloyd George replied, "I propose to deal with this matter in the Finance Bill." Will the *British Medical Journal* now say that no warning had been given? In any case, the broad fact stands out clearly that for many weeks preceding the notice of motion the possibilities of duty-free alcohol for hospitals had been freely and publicly discussed.

The next point is that the opposition of the Association was not to the principle of making a concession to hospitals, but to the method by which this concession was proposed to be made. It is objected that the clause would have based the amount of the grant to a hospital on the amount of alcohol which it had "contrived" to consume, and that the official definition of a hospital opened the door to widespread abuse. What an unhappy choice of a word! What a slander on the voluntary hospital system to suggest that an institution might "contrive" to increase its income by several pounds a year by pouring a few gallons of spirit into the sink, or overdosing its patients with spirituous compounds!

### The Definition of a Hospital.

As to the official definition of a hospital we foresaw possible criticisms, and, dealing with them in advance, in our issue of June 26 pointed out that the definition involved no real danger, since it could safely be left to the Commissioners of Customs and Excise to discriminate between the institutions that were contemplated by the clause and those that were not. Manufacturers who have had occasion to apply for the use of duty-free alcohol in their processes have certainly never had cause to complain of any laxity on the part of the Commissioners, and had the hospital spirit clause passed into law there might have been many complaints of their carefulness, but none of their carelessness. The clause gave wide powers of discrimination to the Board of Customs and Excise, and had those who opposed it been better informed of the methods employed by the Board in protecting the revenue they would have known that the prevention of abuse was in safe hands. But in any case both the objections raised by the Association could have been removed by amendments which would not have affected the principle, and no valid excuse can be given for the point-blank opposition which resulted in the withdrawal of the clause. Our contemporary describes its proposal for "a full and proper inquiry" into the best way of dealing with the subject of duty-free alcohol in medicine before a concession is granted, as a "constructive suggestion."

We remember to this day a "full and proper inquiry" into the question of sewage disposal; it lasted sixteen years, and its recommendations still await the pleasure of Parliament. Finally, the *British Medical Journal* asks "the whole profession, hospitals included," to thank the Association for ensuring that medicine shall be exempt from the new eighteenpenny tax on raw alcohol, and points to this alleged achievement as a promise that "the action of the Association on the further and larger matter will be as fruitful of good." This is a remarkable piece of special pleading. The tax referred to arises out of the war legislation dealing with the sale of immature spirit, and the result of that legislation is that in respect of spirituous preparations "recognised by the Commissioners of Customs and Excise" as articles used for medical purposes, hospitals will be repaid the amount of the surtax. The effect is that hospital authorities will be compelled to keep records they have not been obliged to keep before, and that the surtax of eighteenpence per proof gallon will only be refunded in respect of preparations recognised by the Board of Customs and Excise. In other words, hospitals are in a worse position with regard to the use of spirit than they were before the Association took the action for which it claims the thanks of "the whole profession, hospitals included." It is true that they would have been in a still more unfortunate position if they were not granted a repayment of any part of the surtax, but in view of the very definite statement of the Chancellor of the Exchequer that in placing an extra tax on raw spirit he did not propose to put any fresh duty on medicine it is more than probable that provision would have been made for refunding the amount of the surtax even if the Association had never moved in the matter. But, supposing this concession were entirely due to the efforts of the Association, what thanks can it expect for securing for hospitals a rebate of eighteenpence and resisting the offer of a grant of ten times the amount? And there is still the danger that hospitals may "contrive" to increase their incomes at the rate of eighteenpence on every gallon of proof spirit wasted!

